# Tumoral and paratumoral NK cells and CD8^+^ T cells of esophageal carcinoma patients express high levels of CD47

**DOI:** 10.1038/s41598-020-70771-y

**Published:** 2020-08-18

**Authors:** Zuzana Strizova, Jiri Vachtenheim, Martin Snajdauf, Robert Lischke, Jirina Bartunkova, Daniel Smrz

**Affiliations:** 1 Department of Immunology, Second Faculty of Medicine, Charles University and University Hospital Motol, V Uvalu 84, 150 06 Praha 5, Czech Republic; 2 Third Department of Surgery, First Faculty of Medicine, Charles University and University Hospital Motol, Prague, Czech Republic

**Keywords:** Immunology, Immunosuppression

## Abstract

In a limited number of human malignancies, anti-CD47 therapy leads to the rapid clearance of tumor cells by macrophages. In esophageal squamous cell carcinoma, anti-CD47 treatment has shown promising results in vitro. However, the CD47 expression pattern in tumor-infiltrating lymphocytes (TILs), which are associated with prolonged overall survival and serve as a positive prognostic factor, is largely unknown. In this study, a total of 36 tissue samples from the tumor, peritumoral tissue, and adjacent healthy esophageal tissue was obtained from 12 esophageal carcinoma (EC) patients, and the surface expression of CD47 was evaluated in natural killer (NK) cells, CD8^+^ T cells, and the nonlymphocyte cell fraction. We found that the proportions of the evaluated cells and their CD47-expressing populations were comparable across the analyzed tissue compartments. However, the proportions of CD47-expressing populations in the analyzed tissue compartments were significantly higher in NK cells and CD8^+^ T cells than in the nonlymphocyte cell fraction. Importantly, the intensity of CD47 staining was also significantly higher in the tested immune cells than in the nonlymphocyte cell fraction. High expression of CD47 in tissue-infiltrating NK cells and CD8^+^ T cells in EC patients can, therefore, affect the efficacy of anti-CD47 therapy in EC.

## Introduction

CD47 is a widely expressed transmembrane protein of the immunoglobulin superfamily^1^. Known as a myeloid-specific immune checkpoint^2^, CD47 has been shown to be involved in multiple T cell functions, regulating either the attenuation or enhancement of T cell responses^3^. CD47 interacts with several molecules such as trombospondin 1 (TSP-1), integrins, and signal regulatory proteins *γ* (SIRPγ) and α (SIRPα)^1^. SIRPα is predominantly expressed on macrophages, monocytes, granulocytes, and dendritic cells^4^. Its interaction with CD47 induces an inhibitory “don’t eat me” signal that prevents cells from phagocytosing CD47-expressing cells^1^.

CD47 is also expressed in tumor cells in a number of human malignancies^3^. CD47 overexpression is associated with a poor prognosis in bladder cancer^5^, breast cancer^6^, and different types of leukemia^7^ and is considered to be a marker of cancer recurrence^6^. Since CD47 prevents the phagocytosis of tumor cells^1^, CD47 antagonists have been tested in cancer immunotherapy^8^. Currently, there are several ongoing phase I clinical trials testing these antagonists for the treatment of solid and hematological malignancies^9,10^. An anti-CD47 antibody would presumably increase tumor cell phagocytosis and antitumor CD8^+^ T cell response priming^11^. However, the detailed mechanism underlying the treatment efficacy of anti-CD47 therapy is still unknown.

Esophageal carcinoma (EC) remains one of the most lethal human malignancies, with a 5-year survival rate of less than 15%^12^. CD47 is overexpressed in the tumor tissues of esophageal squamous cell cancer (ESCC) patients^13,14^. In combination with other molecules, CD47, therefore, represents a prognostic factor in ESCC^14^. In vitro experiments have shown that blocking CD47‑SIRPα signaling with anti‑CD47 antibodies increases the phagocytosis of CD47-expressing ESCC tumor cells by macrophages in a dose-dependent manner^13^. These findings indicate that anti-CD47 therapy could be an effective treatment modality for ESCC^13^.

Apart from the tumor cells, lymphocytes are present in EC tumors (TILs, tumor-infiltrating lymphocytes). In EC, increased numbers of TILs have been positively associated with a favorable prognosis^15–17^. However, it is not known whether TILs in EC also express CD47, which would mark these cells as targets for anti-CD47 immunotherapy.

In this study, we aimed to analyze the expression of the CD47 molecule in both tumor-infiltrating lymphocytes and the nonlymphocyte cell fraction of tumoral and paratumoral tissue samples from EC patients. We evaluated 36 tissue samples of 3 different tissue compartments, the tumor, peritumoral tissue, and adjacent healthy esophageal tissue, obtained from 12 esophageal carcinoma patients. Using flow cytometry, we determined the expression of CD47 in NK cells, CD8^+^ T cells, and the nonlymphocyte cell fraction.

## Results

### The proportions of NK cells and CD8^+^ T cells are comparable between the tumoral and paratumoral tissue compartments

**Figure 1:**
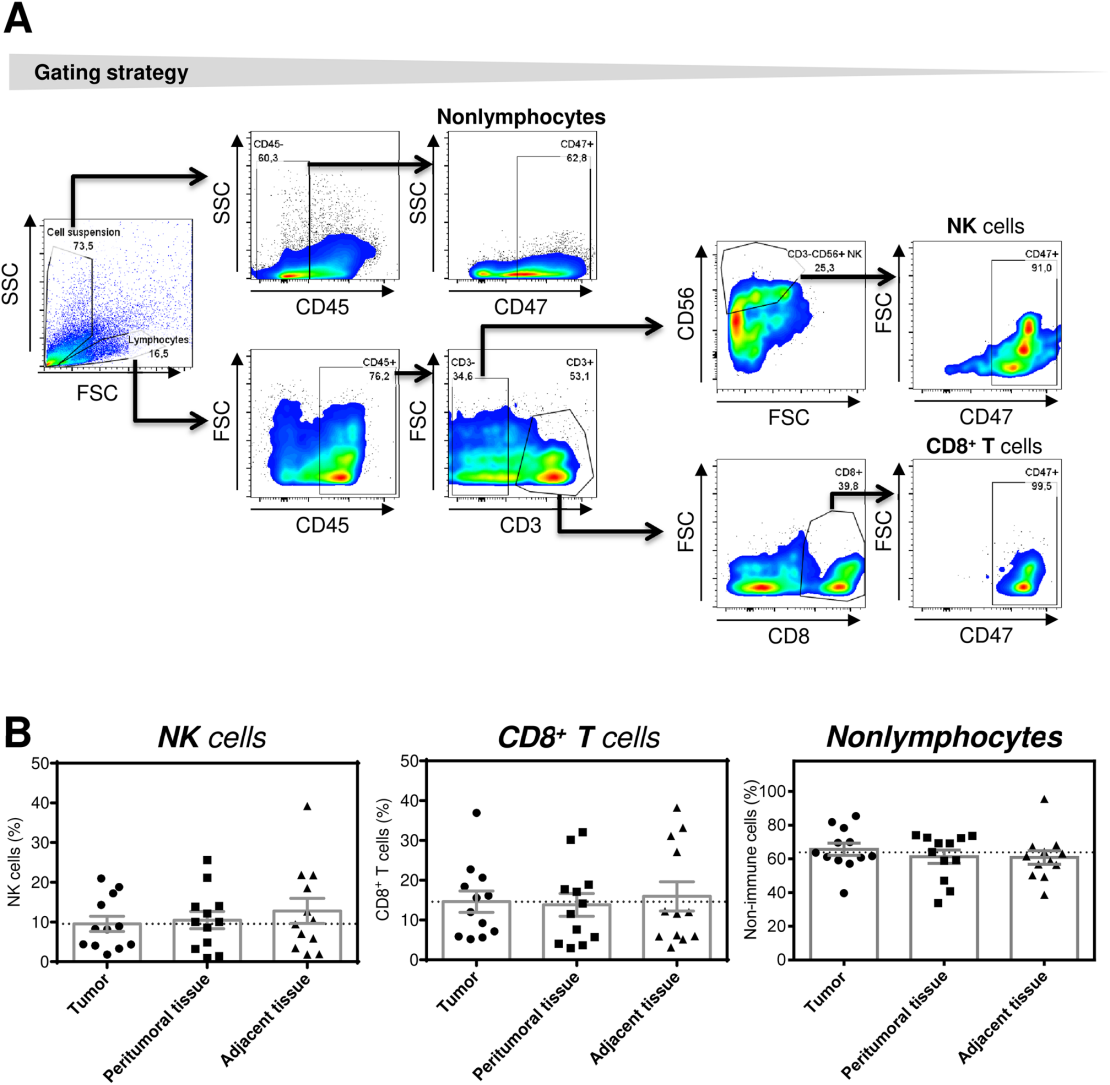
Proportions of NK cells, CD8^+^ T cells, and nonlymphocytes in the tumoral and paratumoral compartments of EC patients. (**A**) The gating strategy for flow cytometry analyses is shown. (**B**) The analyzed cells were gated as in (**A**), and the proportions of NK cells (CD45^+^CD3^−^CD56^+^), CD8^+^ T cells (CD45^+^CD3^+^CD8^+^), and nonlymphocytes (CD45^−^) in tumor tissue, peritumoral tissue, and adjacent tissue samples from 12 EC patients were determined by flow cytometry. The significance of differences was determined by nonparametric one-way ANOVA with Dunn's multiple comparison test (*n* = 12). *P < 0.05 was considered significant. The data are shown as the mean ± SEM.

In this study, 36 tissue samples from 12 patients who underwent surgery for EC were evaluated (Table 1). The tissue samples were obtained from tumor tissue, peritumoral tissue, and adjacent healthy esophageal tissue. The tissue samples were dissociated, and the isolated cells were stained with antibodies specific to CD45, CD3, CD8, and CD56; analyzed by flow cytometry; and evaluated according to the gating strategy shown in Fig. 1A. As shown in Fig. 1B, no significant differences were found in the proportions of NK cells (CD45^+^CD3^−^CD56^+^ cells), T cells (CD45^+^CD3^+^CD8^+^ cells), or the nonlymphocyte population (CD45^−^ cells) among the analyzed tissue compartments (Fig. 1B). These data showed that compared with paratumoral tissues (peritumoral and adjacent healthy tissues), the analyzed tumors were not infiltrated with more NK cells or CD8^+^ T cells.

**Table 1:**
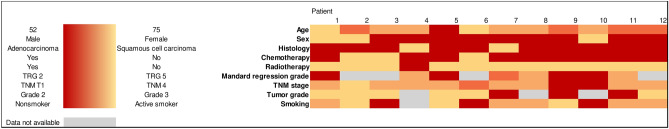
Clinical data heat map.

### Tumoral and paratumoral NK cells and CD8^+^ T cells are highly positive for CD47

**Figure 2:**
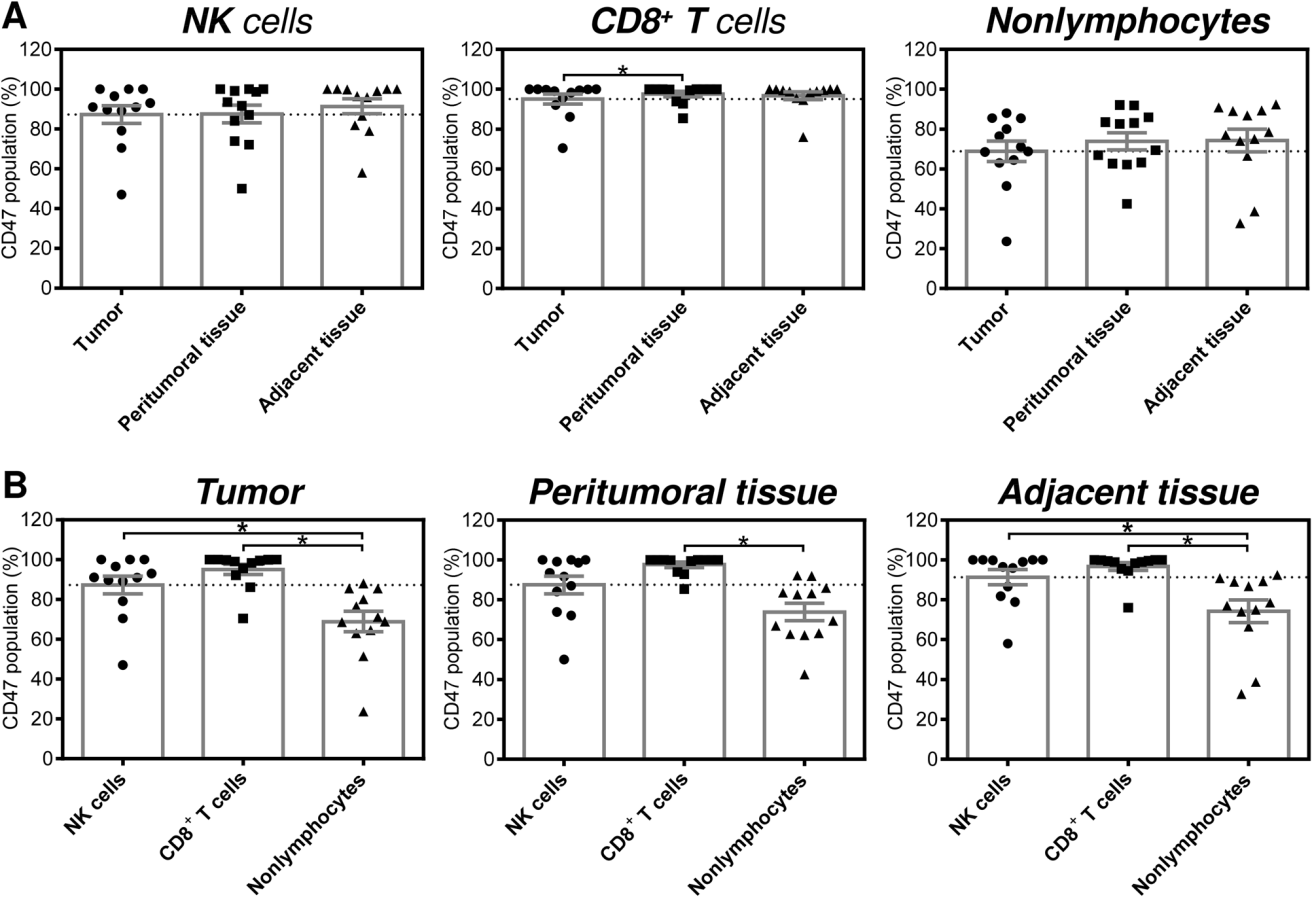
Proportions of CD47-expressing NK cells, CD8^+^ T cells, and nonlymphocytes in the tumoral and paratumoral compartments of EC patients. (**A**) The analyzed cells were gated as in Fig. 1A, and the proportions of CD47-expressing NK cells (CD45^+^CD3^−^CD56^+^CD47^+^), CD8^+^ T cells (CD45^+^CD3^+^CD8^+^CD47^+^), and nonlymphocytes (CD45^−^CD47^+^) in tumor tissue, peritumoral tissue and adjacent tissue samples from 12 EC patients were determined by flow cytometry. (**B**) The proportions of cells in (**A**) were evaluated within each tissue compartment. In (**A**) and (**B**), the significance of differences was determined by nonparametric one-way ANOVA with Dunn's multiple comparison test (*n* = 12). *P < 0.05 was considered significant. The data are shown as the mean ± SEM.

In the next analysis, we evaluated the proportions of CD47-expressing populations of NK cells and CD8^+^ T cells in the tested compartments. As shown in Fig. 2A (two left panels), both cell types were highly positive for CD47, and no significant differences were observed among the tested compartments. Additionally, no significant differences among the compartments were observed for the nonlymphocytes (Fig. 2A, right panel). However, the data indicated that the proportions of CD47-expressing NK cells and CD8^+^ T cells were higher than those of the corresponding nonlymphocytes. Indeed, the proportion of CD47-expressing CD8^+^ T cells in the peritumoral tissue was significantly higher than the proportion of CD47-expressing nonlymphocytes (Fig. 2B, middle panel). In the tumor and adjacent healthy tissue, significantly higher proportions of CD47-expressing cells were found for both NK cells and CD8^+^ T cells (Fig. 2B, left and right panel).

### Tumoral and paratumoral NK cells and CD8^+^ T cells express more CD47 than their nonlymphocyte counterparts

**Figure 3:**
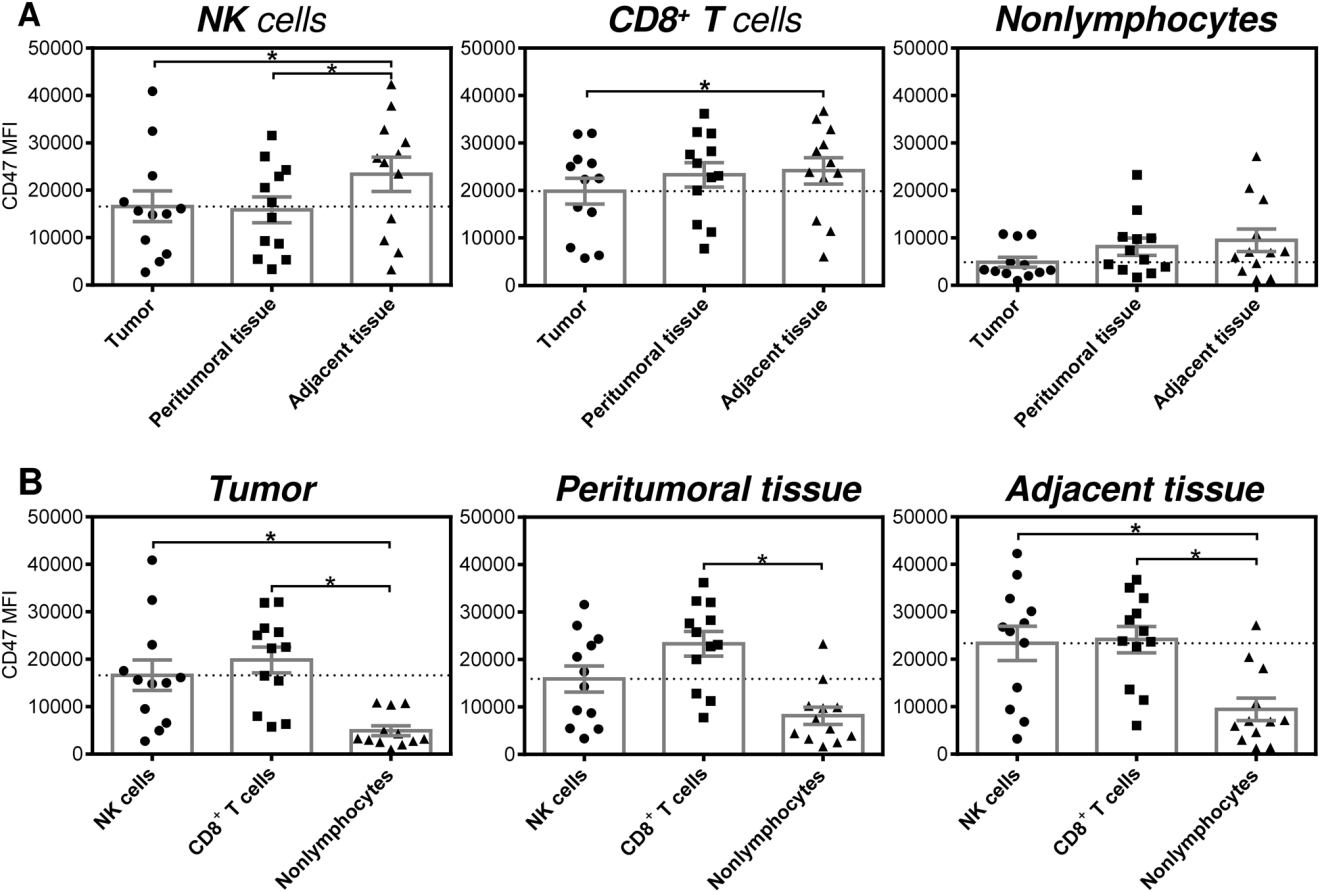
Intensities of CD47 staining of NK cells, CD8^+^ T cells, and nonlymphocytes in the tumoral and paratumoral compartments of EC patients. (**A**) The analyzed cells were gated as in Fig. 1A, and the mean fluorescence intensities of CD47 staining (CD47 MFI) of NK cells (CD45^+^CD3^−^CD56^+^), CD8^+^ T cells (CD45^+^CD3^+^CD8^+^), and nonlymphocytes (CD45^−^) in tumor tissue, peritumoral tissue and adjacent tissue samples from 12 EC patients were determined by flow cytometry. (**B**) The CD47 MFIs determined in (**A**) were evaluated within each tissue compartment. In (**A**) and (**B**), the significance of differences was determined by nonparametric one-way ANOVA with Dunn's multiple comparison test (*n* = 12). *P < 0.05 was considered significant. The data are shown as the mean ± SEM.

The evaluated flow cytometry data indicated that apart from the differences in the proportions of CD47-expressing populations among the tested cell types, the fluorescent intensities of their CD47 staining were also different (Fig. 1A). Therefore, we compared the intensities of NK cells, CD8^+^ T cells, and nonlymphocytes in the 3 tested compartments. The data showed that the NK cells and CD8^+^ T cells in the tumor had significantly lower staining intensity for the CD47-specific antibody than their counterparts in the adjacent healthy tissue (Fig. 3A, two left panels). Comparable results were also observed for the peritumoral NK cells (Fig. 3A, left panel). Unlike the tested immune cells, the nonlymphocytes showed nonsignificant changes in CD47 staining, although the tumoral population showed a tendency toward decreased CD47 staining intensity (Fig. 3A, right panel). Significant differences were, however, found when the CD47 staining intensities were compared among the tested cell types in each individual compartment. As shown, the tumor- and adjacent tissue-infiltrating NK cells and CD8^+^ T cells exhibited significantly higher staining intensity with the CD47-specific antibody than the nonlymphocyte population (Fig. 3B, left and right panels). In the peritumoral tissue, only the CD8^+^ T cells exhibited a significantly increased staining intensity (Fig. 3B, middle panel).

### NK cells stimulation is not impacted by anti-CD47 antibody

**Figure 4:**
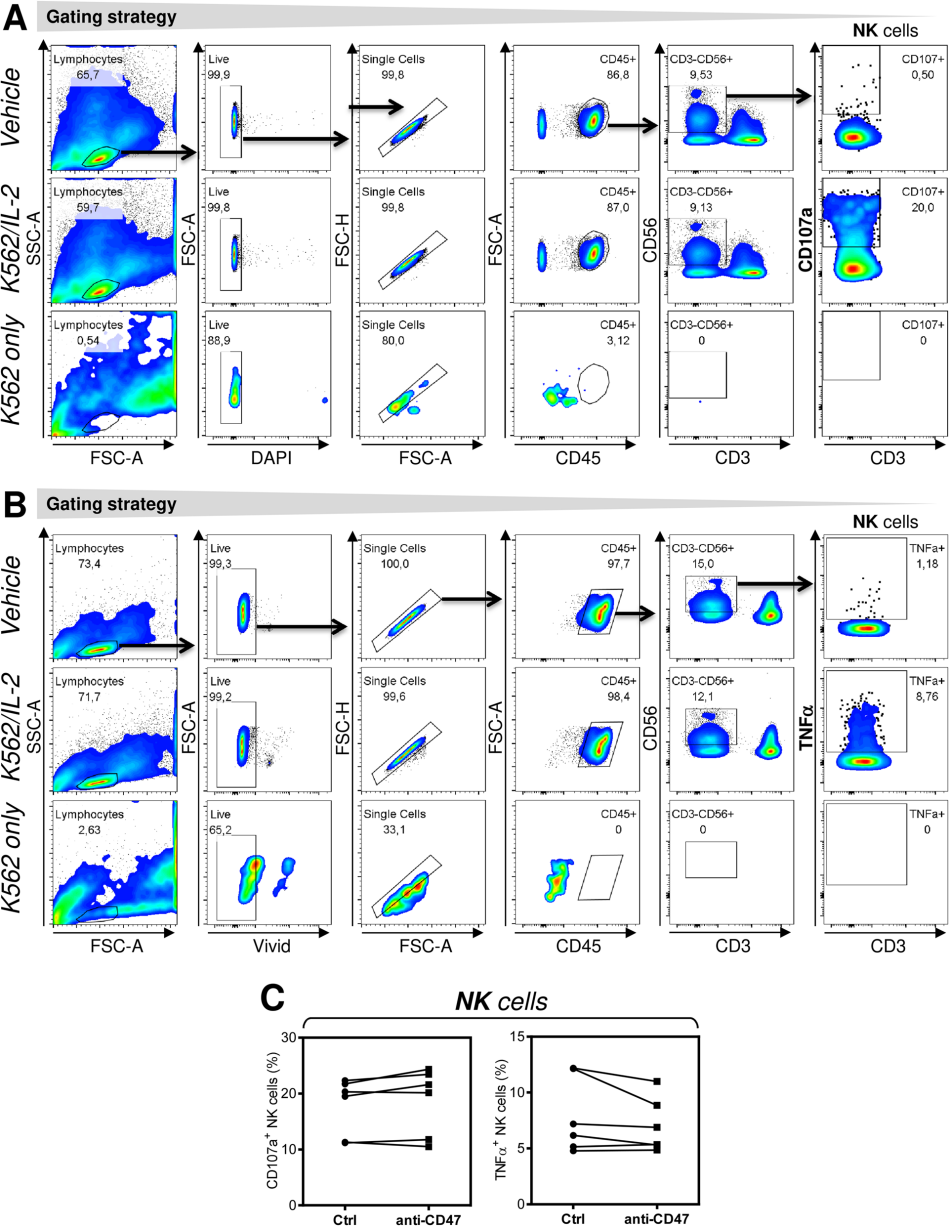
NK cell stimulation is not impacted by anti-CD47 antibody. (**A**) The gating strategy for flow cytometry analyses of CD107a externalization in NK cells (CD45^+^CD3^−^CD56^+^). PBMCs were treated or not treated with anti-CD47 antibody and stimulated with vehicle (Vehicle, top panels) or K-562 cells and IL-2 (K562/IL-2, middle panels). As a control, the target K-562 cells alone were analyzed (K562 only). (**B**) The gating strategy for flow cytometry analyses of TNFα intracellular staining in NK cells (CD45^+^CD3^−^CD56^+^). The samples as in (**A**) are show. (**C**) The data in (**A**) and (**B**) were evaluated and the significance of differences determined by Wilcoxon matched-pairs signed-ranks test (*n* = 6 donors). *P < 0.05 was considered significant.

A previous report has shown that anti-CD47 antibody increased T cell-mediated cytotoxicity when incubated with the effector and target cells^18^. We investigated whether targeting of CD47 on NK cells with specific antibody could impact functionality of NK cells after their stimulation with the target cells and IL-2. As shown in Fig. 4, pretreatment of PBMCs with anti-CD47 antibody for 18–20 h had no impact on cytotoxicity (Fig. 4A,C) and inflammatory response (Fig. 4B,C) of NK cells. The data suggested that CD47 on the surface of NK cells does not impact the stimulation of NK cells in vitro once challenged with anti-CD47 antibody.

## Discussion

CD47 is often overexpressed in many tumors. This molecule has therefore become a target molecule for cancer therapy. In this study, we show that in addition to tumor cells, the majority of tumoral and paratumoral NK cells and CD8^+^ T cells also express CD47, and the levels of its expression are even higher than in the parallel nonlymphocyte cells. These findings indicate that these immune cells will also be targeted upon anti-CD47 therapy.

The expression of CD47 on the cell surface is generally considered as a “don’t eat me” signal^1^. Our findings showed that tumoral and paratumoral NK cells and CD8^+^ T cells of EC patients express high levels of CD47 on their surface. These findings suggest that NK cells and CD8^+^ T cells may use CD47 as the signaling molecule to evade elimination by the infiltrating macrophages. Interestingly, CD47 expression levels on NK cells and CD8^+^ T cells were decreased in the tumor compartment. This decrease could relate to their increased cytotoxic activity^19,20^. In vitro experiments indeed showed that forced downregulation of CD47 or its targeting by anti-CD47 antibody in CD8^+^ T cells increased their cytotoxic activity^18^. Our data, however, did not confirm an increase in the cytotoxic and inflammatory response of stimulated NK cells after their treatment with anti-CD47 antibody. This indicates that the antibody-mediated targeting of CD47 may not equally impact the functionality of NK cells and CD8^+^ T cells. Regardless of this, the levels of CD47 on the surface of the tested NK cells and CD8^+^ T cells were mostly higher than in the parallel nonlymphocyte cell fraction, which contains tumor cells in the tumoral compartment. This indicates that these immune cells will be relatively efficiently impacted by anti-CD47 immunotherapy.

Anti-CD47 immunotherapy relies on monoclonal antibodies that bind to CD47, which is expressed on the cell surface^10^. Binding of monoclonal antibodies to CD47 relieves the block that prevents the cells from being phagocytosed by macrophages^21^. Antibody binding can, however, also trigger cell apoptosis^22,23^. Whether anti-CD47 antibodies mediate macrophage-mediated phagocytosis of the bound cells or trigger apoptosis in the bound cells depends on the type of targeted cell and the type and concentration of the antibody used^13,21^. CD47-specific antibodies have been shown to trigger apoptosis in tumor cells^22,23^ and immune cells^24^. Whether the use of anti-CD47 immunotherapy would also trigger apoptosis in CD47-expressing populations of tumoral and paratumoral NK cells and CD8^+^ T cells in EC patients or only alter the effector functions of these immune cells^25^ needs to be determined. However, what can already be presumed is that anti-CD47 immunotherapy will inhibit the CD47-mediated “don’t eat me” signal^10,13^. As such, unless these immune cells provide another “don’t eat me” signal that surpasses the impact of the antibody binding to CD47, these cells can be destined for elimination by infiltrating macrophages^13,26^.

On the other hand, blocking CD47 can also prevent the augmentation of Fas/CD95-mediated apoptosis in TILs^27^. This mechanism can be used by tumors to evade elimination by TILs^28^. Whether this mechanism also comes into play in EC is not currently known. However, regardless of which of these mechanisms prevail in infiltrating NK cells and CD8^+^ T cells, anti-CD47 immunotherapy in EC will modulate the tumor immune microenvironment^29^.

Infiltration of EC tumors with lymphocytes is associated with a better prognosis and prolonged overall survival^15–17^. The findings of this study indicate that the administration of anti-CD47 immunotherapy can impact the tumoral and paratumoral infiltrating lymphocytes of EC patients. Whether this impact would be beneficial or detrimental to the efficacy of anti-CD47 immunotherapy in EC is difficult to predict. However, this impact needs to be taken into account when anti-CD47 immunotherapy is considered in EC.

In this study, we showed that the tumoral and paratumoral NK cells and CD8^+^ T cells of EC patients express high levels of CD47 on the cell surface. This can significantly contribute to the efficacy of anti-CD47 immunotherapy in EC.

## Materials and methods

### Patients and tissue samples

In this study, 36 tissue samples from 12 patients were included. The patients underwent a planned surgery between September 2018 and March 2019 at the Third Department of Surgery, First Faculty of Medicine, Charles University and University Hospital Motol in Prague. The age of the patients ranged from 52 to 75 years (mean 66.8 years). After surgery, 3 tissue samples were resected from 3 tissue compartments: the tumor, peritumoral tissue, and adjacent healthy esophageal tissue. This study was approved by the Ethics Committee of the University Hospital Motol in Prague (No. EK-810/18), and all patients provided signed written informed consent to participate in the study.

### Cell isolation

Resected tissue samples were immediately transferred into a container with 5 ml of RPMI 1640 medium (Thermo Scientific, Waltham, MA) and immediately transported for processing. The tissues were mechanically dissociated (approximately 1 mm in diameter), and then cells isolated as described previously^30^.

### Flow cytometry

Isolated cells were stained with fluorophore-conjugated antibodies as described previously^31^. The antibodies were anti-CD45 AF700 (Exbio, Prague, Czech Republic), anti-CD3 PerCP-Cy5.5 (Thermo Scientific), anti-CD56 FITC (Exbio), and anti-CD47 APC (BioLegend, San Diego, CA). The isolated cells were washed and then analyzed with a FACSAria II flow cytometer (Becton Dickinson, Franklin Lakes, NJ). The acquired data were analyzed by FlowJo Software (Tree Star, Ashland, OR). The gating strategy is shown in Fig. 1A.

### CD107a assay and intracellular staining of NK cells

Peripheral blood was obtained from 6 healthy volunteers (the age of the donors ranged from 31 to 44 years (mean 36.5 years). Peripheral blood mononuclear cells (PBMCs) were isolated as previously described^31^. The fresh-isolated cells were transferred to a culture medium [RPMI 1640 medium, 5% human plasma serum (One Lambda, Canoga Park, CA), 100 U/ml penicillin–streptomycin, 2 mM Glutamax, 1 mM sodium pyruvate and nonessential amino acid mix (Thermo Scientific)] and cultured at a concentration of 2 × 10^6^ cells/ml in the presence or absence of CD47-specific antibody (20 μg/ml; clone MIAP410, InVivoMab, BioXCell, Lebanon NH) for 18–20 h. The cells were harvested, pelleted, and washed twice with the culture medium. For the CD107a assay, 350 × 10^3^ cells in 100 μl were transferred to a 96 V-bottom well plate (Nalgene, Rochester, NY). The wells were supplemented with 100 μl of culture medium alone or containing 35 × 10^3^ of K-562 cells and 20,000 IU/ml of IL-2 (Peprotech, Rocky Hill, NJ). The plate was centrifuged at 30×*g* for 3 min at room temperature and then incubated for 2 h at 37 °C and 5% CO_2_. The cells were stained as above using the anti-CD45 AF700 (Exbio), anti-CD3 PerCP-Cy5.5 (Thermo Scientific), and anti-CD56 FITC and anti-CD107a PE (Exbio) antibodies. For the intracellular staining, the cells were stimulated in a 96 U-bottom well plate (Nalgene) as above. After 1 h of stimulation (37 °C, 5% CO_2_), the cells were supplemented with brefeldin A (BioLegend, San Diego, CA) and then cultured for 4.5 h. The cells were transferred to the V-bottom 96-well plate, stained with live/dead fixable stain, fixed, and permeabilized as previously described^32^. The cells were then stained with the anti-CD45 AF700, anti-CD3 PerCP-Cy5.5, anti-CD56 FITC, and anti-TNFα APC (Becton Dickinson) antibodies. The stained cells were analyzed by flow cytometry to determine the NK cell cytotoxic (CD107a expression^33^) and inflammatory (TNFα production) response.

### Statistical analysis

Means and SEMs were calculated from the indicated sample size (*n*) using GraphPad Prism 6 (GraphPad Software, La Jolla, CA). Statistical significance between three or more groups was determined by nonparametric one-way ANOVA with Dunn's multiple comparison test. Statistical significance between two groups of differentially treated samples was determined by Wilcoxon matched-pair signed-rank tests. *P* < 0.05 was considered significant.

### Ethical approval

This study was approved by the ethics committee for multicentric studies and evaluation of the University Hospital Motol, Prague, Czech Republic. All procedures in the studies involving human participants were performed in accordance with the ethical standards of the institutional and/or national research committee and with the 1964 Helsinki declaration and its later amendments or comparable ethical standards.

### Informed consent

Informed consent was obtained from all individual participants included in the study.
